# Studies on Oxygen Permeation Resistance of SiCN Thin Film and RRAM Applications

**DOI:** 10.3390/nano12234342

**Published:** 2022-12-06

**Authors:** Myeong-Ho Song, Woon-San Ko, Geun-Ho Kim, Dong-Hyeuk Choi, Ga-Won Lee

**Affiliations:** 1 Nano Convergence Technology Division, National NanoFab Center, Daejeon 34141, Republic of Korea; 2 Division of Electronics Engineering, Chung-nam National University, Daejeon 34134, Republic of Korea; 3 ISTE Co., Ltd., 306 Toseong-ro, Hyangnam-eup, Hwaseong-si 18589, Republic of Korea

**Keywords:** SiCN, PECVD, HAST, RRAM, endurance

## Abstract

In this study, a silicon carbon nitride (SiCN) thin film was grown with a thickness of 5~70 nm by the plasma-enhanced chemical vapor deposition (PECVD) method, and the oxygen permeation characteristics were analyzed according to the partial pressure ratio (PPR) of tetramethylsilane (4MS) to the total gas amount during the film deposition. X-ray photoelectron spectroscopy (XPS), Fourier transform infrared spectroscopy (FT-IR), and X-ray reflectivity (XRR) were used to investigate the composition and bonding structures of the SiCN film. An atomic force microscope (AFM) was used to examine the surface morphology of the SiCN films to see the porosity. The analysis indicated that Si–N bonds were dominant in the SiCN films, and a higher carbon concentration made the film more porous. To evaluate the oxygen permeation, a highly accelerated temperature and humidity stress test (HAST) evaluation was performed. The films grown at a high 4MS PPR were more susceptible to oxygen penetration, which changed Si–N bonds to Si–N–O bonds during the HAST. These results indicate that increasing the 4MS PPR made the SiCN film more porous and containable for oxygen. As an application, for the first time, SiCN dielectric film is suggested to be applied to resistive random access memory (RRAM) as an oxygen reservoir to store oxygen and prevent a reaction between metal electrodes and oxygen. The endurance characteristics of RRAM are found to be enhanced by applying the SiCN.

## 1. Introduction

Silicon carbon nitride (SiCN) thin films have been researched as one of the alternatives for silicon nitride (SiN) which is used as a diffusion barrier in Cu interconnect technology. SiCN is more favorable as a Cu barrier because it can also solve the resistor–capacitor (RC) delay problem in metal interconnection due to a lower dielectric constant than SiN [1,2]. The barrier characteristics of SiCN mean there is a low permeation rate when the film is exposed to the out-diffusion of copper or inward permeation of water or oxygen. Specifically, oxygen can oxidize the metal line and cause drastic performance degradation, including decreasing device reliability [3,4,5]. As the application range of electronic devices is expanding rapidly and products and services are related to human life, ranging from cars to national defense, the reliability of electronic devices has become more crucial.

In order to evaluate the characteristics of SiCN thin film for oxygen permeation, highly accelerated stress tests (HAST) of temperature and humidity were performed. HAST is a method to check an electronic component’s reliability by evaluating the shortened lifespan in an environment including oxygen. The environment in which the moisture permeability of semiconductor devices is increased with pressurized steam is used [6].

As a new application of SiCN, the film is applied to resistive random access memory (RRAM). In recent studies, RRAM attracted attention because of its nonvolatile characteristics and simple structure that can increase device integration, and it is being studied by applying it in energy storage devices [7]. The operating mechanism of RRAM is based on the formation of conductive filaments via oxygen vacancies generated by the relocation of oxygen ions. Due to the randomness of oxygen movement, the parameter variation is large and must be solved for high-density integration and mass production of RRAM [8,9]. With the variability, reliability is one of the most challenging issues. As in the Cu metal line, oxygen ions migrate to the anode electrode and cannot be combined with its vacancy again by making fixed bonds with the metal electrodes in the RRAM operation, causing instability problems [10,11]. To improve the RRAM reliability, SiCN is suggested as an oxygen reservoir between the metal electrode and resistance-switching layer for the first time. SiCN thin film has been reported as a resistance-switching layer of RRAM [12,13,14,15]. SiCN as an oxygen reservoir, however, has not been researched yet. In this experiment, ZnO-based RRAM, known to have weaker endurance than HfO_2_-based RRAM, was used. The endurance characteristics of RRAM were expected to improve due to the application of a SiCN dielectric thin film to store the oxygen ions.

## 2. Materials and Methods

In this study, SiCN was grown by plasma-enhanced chemical vapor deposition (PECVD), and the characteristics of SiCN were analyzed according to the process conditions of tetramethylsilane (4MS) partial pressure ratio (PPR). SiCN films were deposited on Si (100) substrates with ISTE’s PECVD equipment (ISTE. Hwaseong-si. Korea). Four gases—4MS, ammonia (NH_3)_, helium (He), and nitrogen (N_2)_—were used. The process temperature was set at 350 °C. The pressure was 4.2 Torr, and the plasma power was 650 watts. In the experiments, the 4MS PPR varied from 3% to 12%. Table 1 summarizes the process conditions.

X-ray photoelectron spectroscopy (XPS), Fourier transform infrared spectroscopy (FT-IR), X-ray reflectivity (XRR), and atomic force microscope (AFM) were used to investigate the composition, bonding structures, and surface morphology of the SiCN films. The source of XPS excitation energy was Al Ka line(1486eV), and the XPS energy resolution was equal to or less than 0.5 eV measured at the full-width half-maximum (FWHM). In order to evaluate the characteristics of the SiCN thin film for oxygen permeation, HAST was performed, exposing the film to a high temperature and high humidity environment. HAST conditions were 130 °C, 85% relative humidity, 122 KPa, 96 hours in high humidity, and high-pressure test equipment (EHS-221MD).

Before and after HAST, the concentrations of carbon, nitrogen, oxygen, and silicon were observed using XPS (ThermoFisher Scientific. Waltham, MA, USA) depth profile analysis by Ar monoatomic sputtering with 2000 eV and 1 mm × 1 mm raster size. FT-IR spectroscopy and XRR were used to investigate the changes in bonding structures of the SiCN films after HAST. An atomic force microscope was used to examine the surface morphology of the SiCN films.

**Figure 1:**
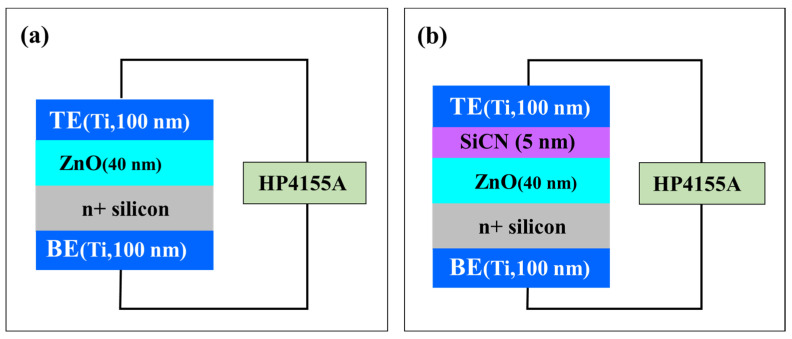
Schematic of RRAM with (**a**) the conventional TE (Ti, 100 nm)/ ZnO (40 nm)/ n+Si/ BE (Ti, 100 nm), and (**b**) the suggested TE (Ti, 100 nm)/ SiCN (5 nm, 4MS PPR 5.2%)/ ZnO (40 nm)/ n+Si/ BE (Ti 100 nm) structure.

The structure shown in Figure 1 was used to evaluate the characteristics of RRAM with the SiCN thin film. First, a 40 nm ZnO thin film was deposited on the N^+^ silicon (100) wafer by the ALD process, and a 5 nm SiCN thin film was deposited thereon by the PECVD process. Next, the SiCN thin film was deposited with the 4MS PPR of 5.2%. Then, 100 nm of titanium (Ti) was deposited as the top electrode (TE) and the bottom electrode (BE) by sputtering.

## 3. Results and Discussion

### 3.1. SiCN Film Properties with 4MS Partial Pressure Ratio

**Figure 2:**
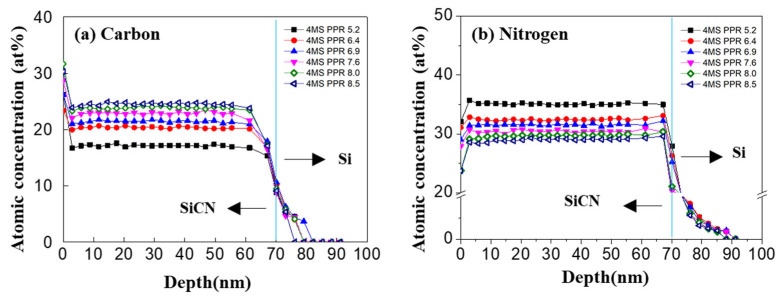

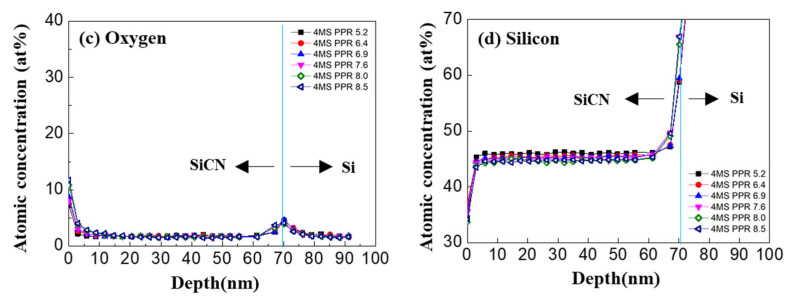
The distribution of components in the depth profile of the thin SiCN film through XPS analysis. (**a**) Carbon (C 1s), (**b**) nitrogen (N 1s), (**c**) oxygen (O 1s), and (**d**) silicon (Si 2p).

In Figure 2, the distributions of carbon, nitrogen, oxygen, and silicon of the thin film were examined in the depth profile by XPS analysis. The concentration of each component in the depth direction of the film was constant. However, the 4MS PPR appeared as an important process factor in the thin film composition change. When the 4MS PPR increased, carbon concentration increased, and nitrogen decreased while oxygen and silicon maintained a constant value. In addition, it was noticeable that the interface with the substrate and SiCN had a higher oxygen concentration of about 2% more than in the thin film and substrate.

**Figure 3:**
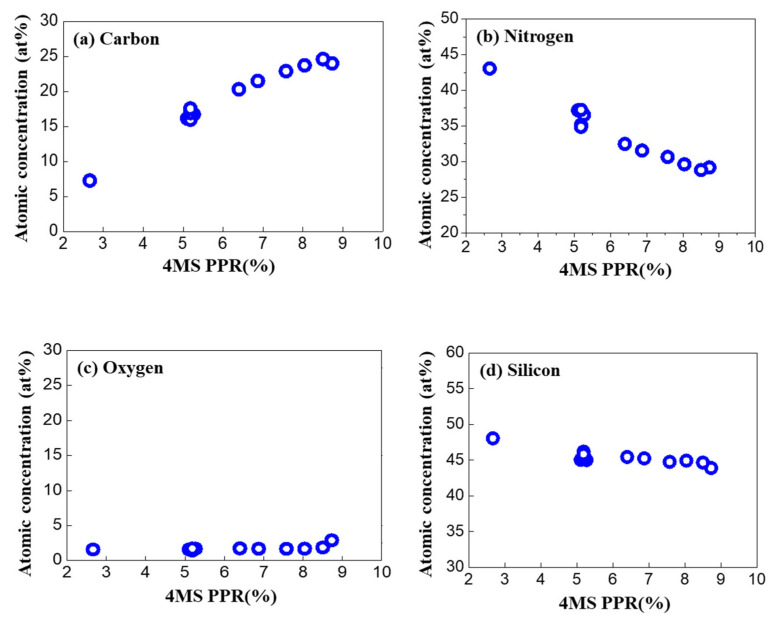
The composition ratio at the one-fourth point of the SiCN thin film according to 4MS PPR. (**a**) Carbon, (**b**) nitrogen (**c**) oxygen, and (**d**) silicon.

Based on the XPS results, the component composition ratio at the one-fourth point (about 20 nm in depth) of the SiCN thin film was compared according to the 4MS PPR, as shown in Figure 3. It was observed that when the 4MS PPR was less than 8%, carbon increased, and nitrogen decreased in proportion to the increase in 4MS PPR. In contrast, oxygen stayed constant without fluctuation. However, different behavior occurred when the 4MS PPR was more than 8%. Carbon and silicon decreased while nitrogen and oxygen increased. 

**Figure 4:**
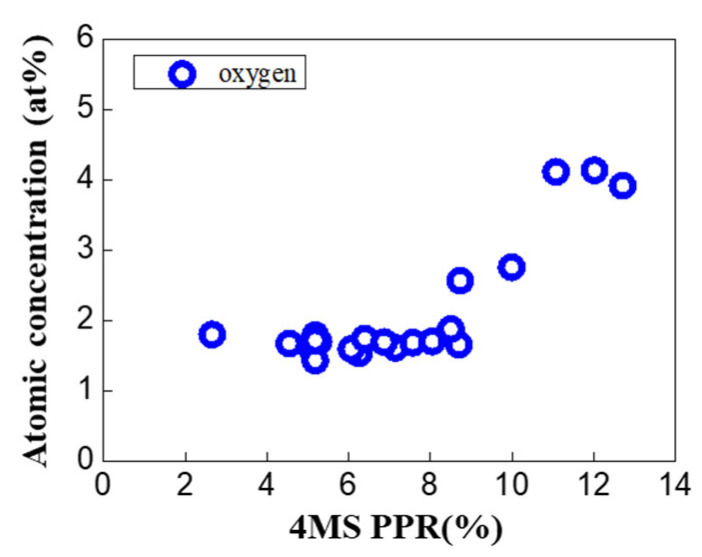
Oxygen concentration according to the 4MS PPR. In this experiment, the 4MS PPR increased to over 8%.

Additional experiments were conducted to confirm the trend of oxygen increase above 8.5% (Figure 3). As a result, it was confirmed that after the deposition of the thin film, the amount of oxygen increased above 8% of the 4MS PPR (Figure 4).

**Figure 5:**
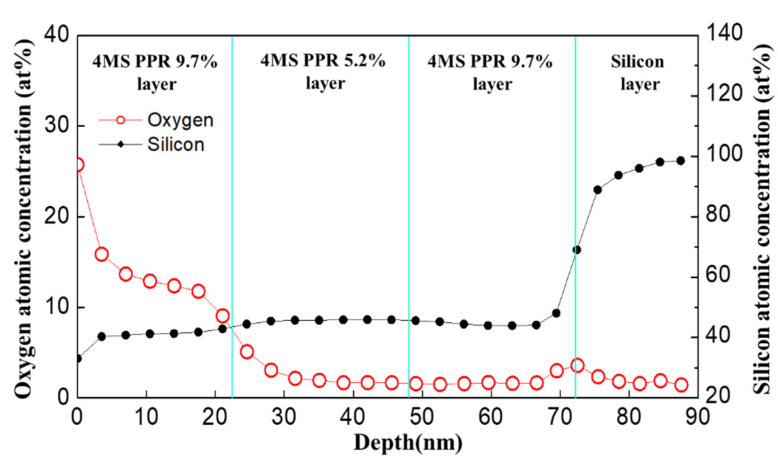
Results of XPS in-depth analysis for three-layered film with 4MS PPR 9.7%, 4MS PPR 5.2%, and 4MS PPR 9.7%.

A three-layered structure was fabricated to find the oxygen source for increasing oxygen concentration by over 8%. The 4MS PPR 9.7% thin film and 4MS PPR 5.2% thin film were mixed to form a sandwich-type of three layers (4MS PPR 9.7% /4MS PPR 5.2% /4MS PPR 9.7%). As shown in Figure 5, the oxygen concentration was higher in the 4MS PPR 9.7% thin film located on the surface than in the 4MS PPR 9.7% thin film located inside. With this result, it was noticed that the film had a low oxygen concentration in the equipment. However, when it was exposed to an oxygen-rich environment, oxygen could penetrate the thin film. It could be inferred that when the 4MS PPR was high, the SiCN thin film had a structure with a low resistance to oxygen permeation. Low resistance to oxygen permeation could degrade the device’s reliability due to poor barrier characteristics.

**Figure 6:**
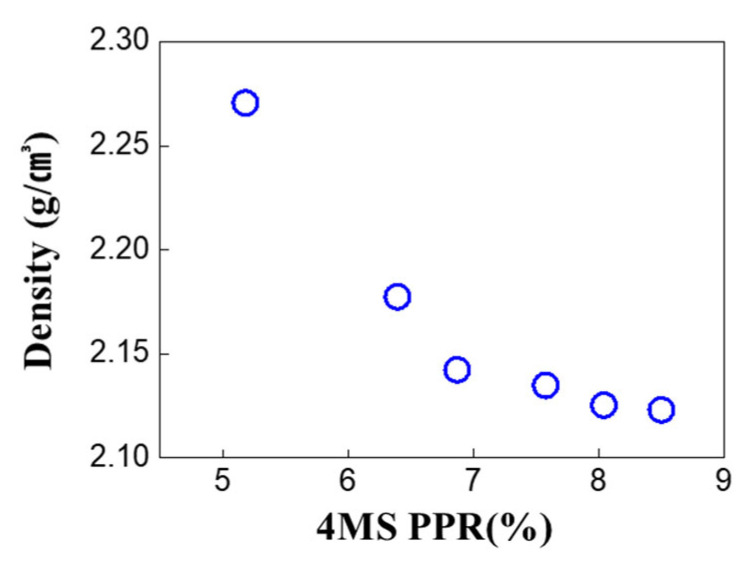
XRR analysis for film density after film deposition.

From XRR analysis, it was observed that as the 4MS PPR increased, the density of the thin film decreased (Figure 6). Lower film density was related to lower resistance to oxygen permeation, which indicated that the film with high porosity could serve as a passage for oxygen transfer into the film (Figure 5) [16,17].

**Figure 7:**
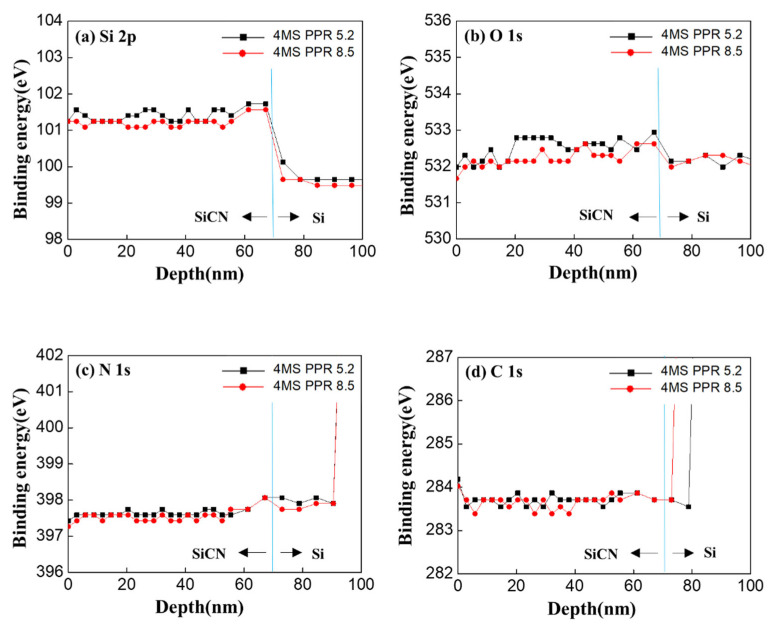
Comparison of binding energies of 4MS PPR of 5.2% and 4MS PPR of 8.5% at the peak of intensity (**a**) Si 2p, (**b**) O 1s, (**c**) N 1s, (**d**) C 1s.

In order to determine what kind of bonding predominated in the SiCN film, changes in the Si bond (Si 2p) in the XPS analysis were analyzed. Peak binding energies were shown for a 4MS PPR of 5.2% and 4MS PPR of 8.5% (Figure 7). As a result, Si–N (101.4~102.2 eV) bonds were mainly found after deposition, and there was no signal in the Si–C bonding region (99.8~100.9 eV) according to Handbook of Compound Type and Binding Energy for XPS. As shown in Figure 7b–d, the binding energies of O 1s, N 1s, and C 1s were distributed at 532~533 eV, 397~398 eV, and 283~284 eV, respectively. Among them, the N 1s binding energy corresponded to Si3N4 and nitride, and the energy range of C 1s was located between the carbon energy range and carbide energy range. [18,19]. Results were the same for Si 2p (Figure 7a). When the depth was over 70 nm, there were no O1s, N 1s, or C 1s peaks except for Si 2p due to a small amount of oxygen, nitrogen, and carbon in the silicon wafer. Thus, the data were not meaningful over 70 nm.

**Figure 8:**
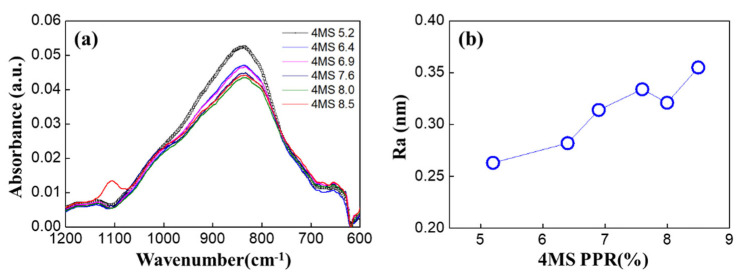
Results of (**a**) FT-IR and (**b**) AFM according to 4MS PPR.

FT-IR analysis was performed to determine the bonds’ changes according to the 4MS PPR. In Figure 8a, as the 4MS PPR increased, 840 cm^−1^ (Si–N, C–H) decreased, and 1100 cm^−1^ (Si–O) increased [20]. The Si–N bond was the main bond in the SiCN thin film, and as the 4MS PPR increased, the Si–N bond decreased. This was consistent with the decreased N concentration with the 4MS PPR increase (Figure 2). In Figure 8b, AFM analysis was performed to study the thin film’s porosity indirectly. As the 4MS PPR increased, the roughness increased, and the film was considered porous, which was consistent with the XRR results in Figure 6.

### 3.2. SiCN Film Properties after a Highly Accelerated Temperature and Humidity Stress Test

**Figure 9:**
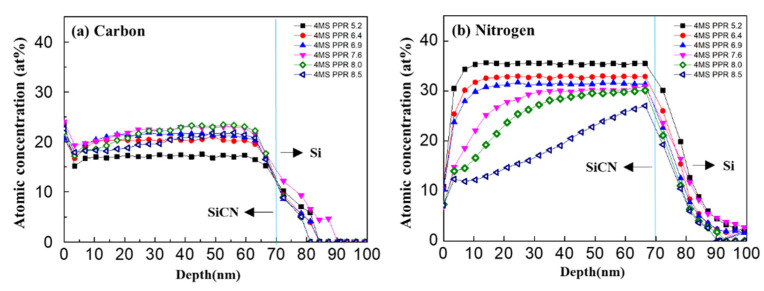

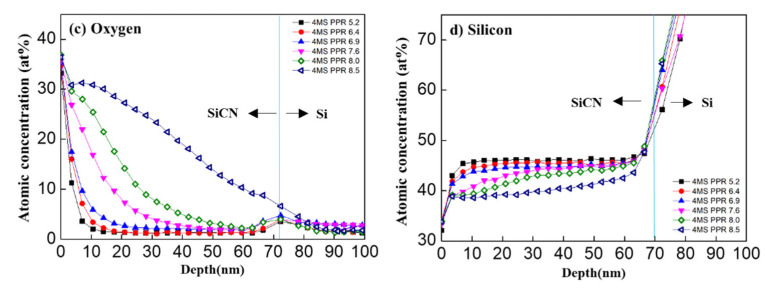
The concentration of components in the depth profile of the thin SiCN film through XPS analysis after HAST. (**a**) Carbon, (**b**) nitrogen, (**c**) oxygen, and (**d**) silicon.

Oxygen penetration could cause reliability problems in electronic devices. Thus, the properties of the SiCN thin film were studied through a HAST. In Figure 9, after the HAST evaluation, it could be seen that oxygen penetration into the thin film took place. At the 4MS PPR of 7.6% or more, oxygen concentration sharply increased, and nitrogen concentration decreased rapidly, suggesting that the decrease in nitrogen according to the increase in oxygen had a mutual effect. Changes in concentration in the depth profile showed a tendency to slope from the surface to the inside of the thin film. Carbon and silicon decreased slightly. These changes after the HAST were presumed to be caused by the formation of an oxygen diffusion path due to increased carbon.

**Figure 10:**
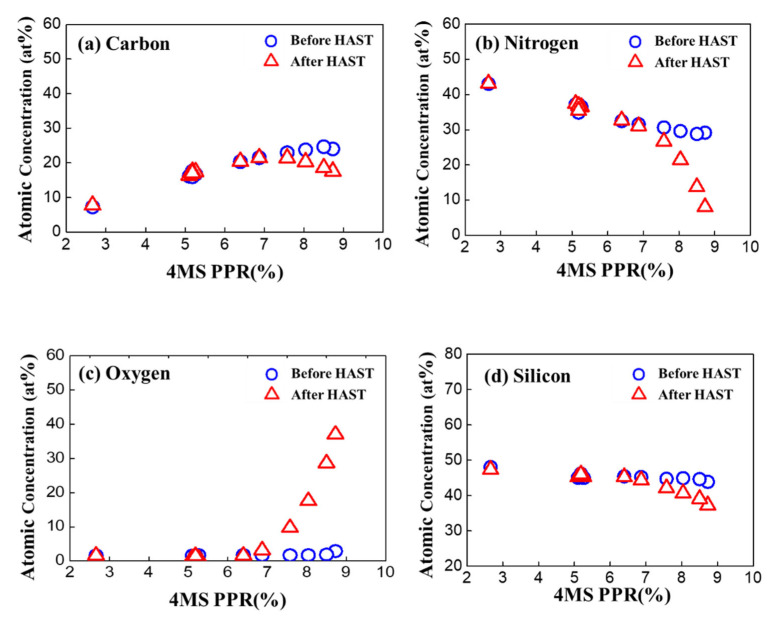
Concentration before and after the HAST according to the 4MS PPS for (**a**) carbon, (**b**) nitrogen, (**c**) oxygen, and (**d**) silicon.

In the graphs of the concentrations at about a 20 nm depth of the SiCN thin film after the HAST, there were no or minor changes when the 4MS PPR was 7% or less (Figure 10). However, when the 4MS PPR was more than 7%, the oxygen concentration significantly increased, and the nitrogen concentration greatly decreased. The concentration of carbon and silicon slightly decreased compared to nitrogen and oxygen. It was shown that when the concentration of carbon in the SiCN thin film was high, the SiCN thin film was vulnerable to oxygen permeation.

**Figure 11:**
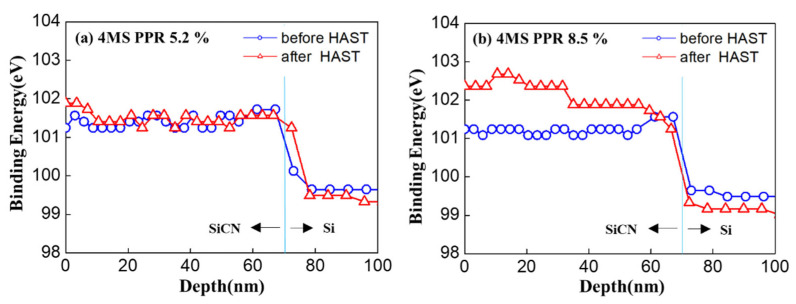
Comparison of the changes in the Si bond (Si2p) in the XPS analysis before and after HAST at (**a**) 4MS PPR of 5.2% and (**b**) 4MS PPR of 8.5%.

In order to determine what kind of bonds were affected by the HAST, the Si bond (Si2p) was analyzed by XPS (Figure 11). It was found that after the HAST, when the 4MS PPR was 5.2%, there were no or minor changes, but when the 4MS PPR was 8.5%, the Si–N (101.4~102.2 eV) bonds changed to Si–N–O (>102 eV) bonds [18,19]. The increased oxygen due to the HAST affected the S–N bond changing to SiN_x_O_y_ or SiO_z_. This result was the same in Figure 10, in which the nitrogen concentration decreased considerably as the oxygen concentration increased significantly after the HAST.

**Figure 12:**
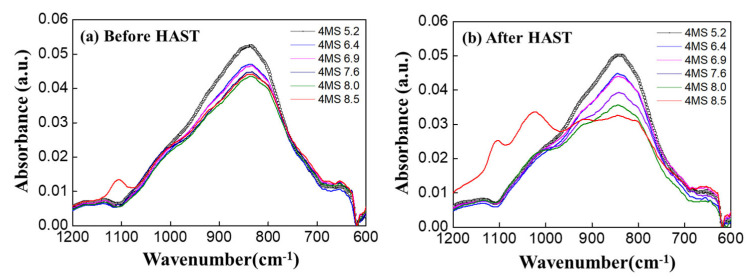
FT-IR analysis results (**a**) before and (**b**) after the HAST according to the 4MS PPR.

In Figure 12a, as the 4MS PPR increased, 840 cm^−1^ (Si–N, C–H) decreased, and 1100 cm^−1^ (Si–O) increased. In Figure 12b, the increase of Si–O (1100, 1020, 920) was demonstrated after the HAST. As the Si–N bond was oxidized, Si–N decreased and the Si–O bond increased, changing to SiNxOy or SiOz, the same as the XPS analysis results in Figure 10.

**Figure 13:**
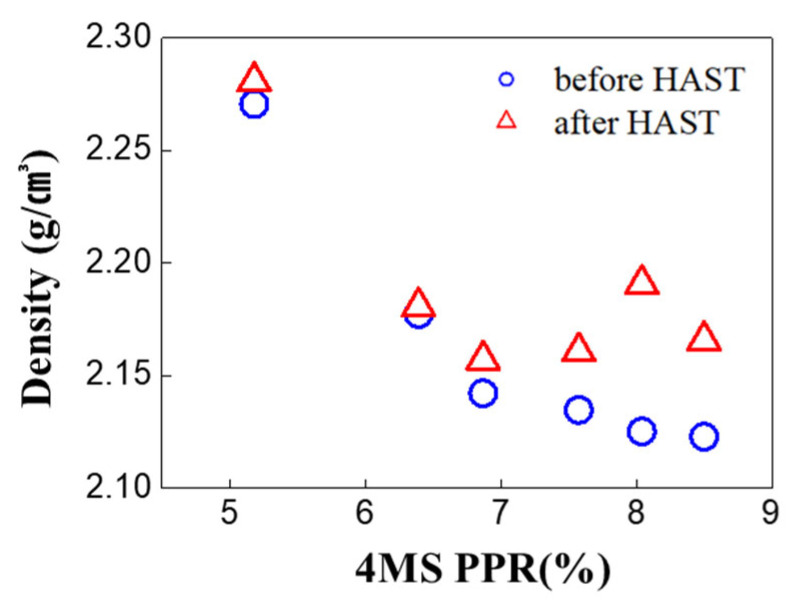
XRR analysis for film density according to the 4MS PPR before and after the HAST.

In Figure 6 of the XRR analysis, it was observed that as the 4MS PPR increased, the density of the thin film decreased. In Figure 13, when the 4MS PPR was 7% or more, the density increased after the HAST. When carbon concentration was high, after the HAST, increases in density were caused by the increase in oxygen concentration through the high porosity which served as a passage for the oxygen movement inside of the film. In SiN_x_ thin film, it has also been reported that some nitride films deposited under porous conditions show oxidation after only a few minutes in the air [21]. It was noticed that through this passage, oxygen moved into the thin film exposed to an external oxygen environment, such as air or a HAST.

**Figure 14:**
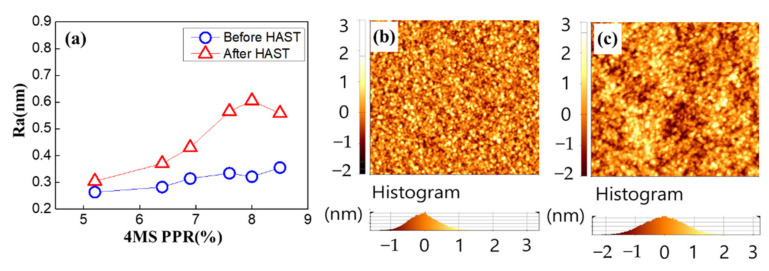
AFM analysis of film roughness (**a**) before and after the HAST. (**b**) Two-dimensional image (1 μm× 1 μm) of 4MS PPR −8.5% as depo. (**c**) Two-dimensional image (1 μm× 1 μm) of 4MS PPR −8.5% after HAST.

Based on the XPS and XRR results, it is inferred that the increase in carbon concentration according to the increase of the 4MS PPR made the thin film porous. The AFM measurement was performed to study the relationship between carbon concentration and porosity [22]. As a result, roughness increased as the 4MS PPR increased, and roughness was saturated at the 4MS PPR more than 7% (Figure 14a). Comparing Figure 14b,c, it was found that the increase in roughness after the HAST was due to regional non-uniformity.

### 3.3. RRAM with SiCN as an Oxygen Reservoir

In RRAM using ZnO, oxygen ions were involved in forming conductive filaments in ZnO combined with the top electrode during the set/reset operation, and the endurance property was degraded [5,23,24]. In order to prevent this degradation, a SiCN thin film (4MS PPR of 5%) was suggested for the first time to block the oxygen ions from combining with the top electrode and served as temporary oxygen storage between the top electrode and ZnO [12,13,14,15].

**Figure 15:**
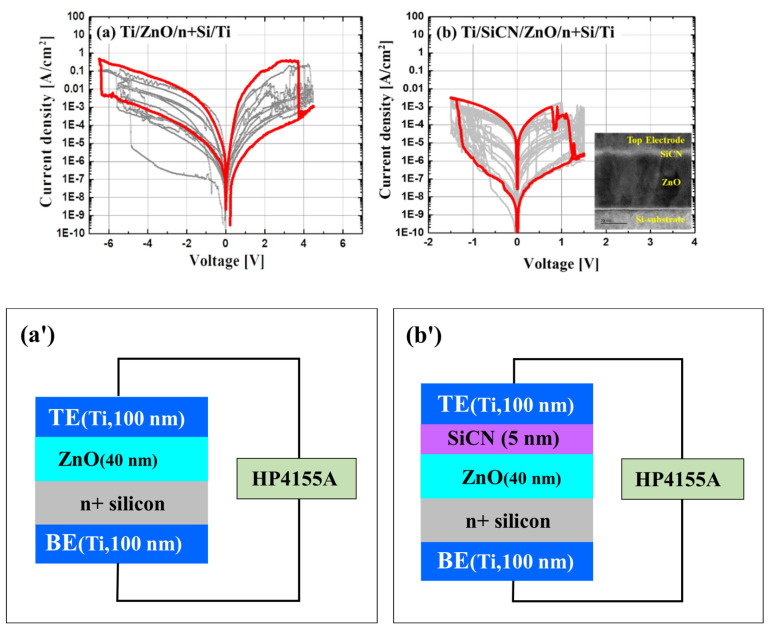
The IV measurements of the fabricated devices with (**a**) ZnO only and (**b**) ZnO/SiCN (**a’**) and (**b’**) schematic section view of (**a**) and (**b**), respectively. Inset of (**b**) is TEM image of RRAM with ZnO/SiCN.

Figure 15 shows the IV measurement results of the fabricated devices with and without the SiCN film as an oxygen reservoir. There was a forming process of −5V to run the traditional RRAM device with only the ZnO layer. However, the suggested RRAM device with the SiCN/ZnO layer seemed to be forming-free; thus, there was no forming process. 

The experimental results showed that the RRAM endurance improved about five times as expected by applying the SiCN thin film, and the characteristics became more stable. The electrical parameters are summarized in Table 2.

## 4. Conclusions

In this study, a SiCN thin film was analyzed, focusing on the oxygen movement-related properties according to process conditions. When forming a SiCN thin film, the partial pressure ratio of 4MS gas in the equipment chamber appeared to significantly affect the thin film’s composition as one of the key process parameters. The increase in the 4MS PPR made the thin film more porous and increased the carbon concentration. Moreover, oxygen could easily permeate the SiCN thin film due to this porous structure. When the SiCN thin film formed with the 4MS PPR of 8% or more, the SiCN absorbed oxygen when exposed to an oxygen environment. It was concluded that the carbon in the SiCN thin film contributed to making a porous structure, and Si–N bonds occupied a significant portion of the thin film. Oxygen migrating into the SiCN thin film was shown to be involved in the Si–N bonding to form SiN_x_O_y_ or SiO_z_. 

As an application of SiCN in electronic devices, the ZnO RRAM with SiCN as an oxygen reservoir was suggested, and the devices were fabricated. The experimental results showed that endurance characteristics could be enhanced using a thin SiCN layer between ZnO and metal electrodes. Applying the SiCN thin film with oxygen-absorbing properties could prevent deterioration caused by the metal electrode and oxygen reaction.

## Figures and Tables

**Table 1:** Process conditions of SiCN films deposited using four gases (4MS, NH_3_, He, and N_2_) by PECVD of VAULER Ⅱ from ISTE (www.iste.co.kr, accessed on 25 September 2022).

4MS PPR	Temp.	Pressure	Power	Gas Flow (sccm)			
	(°C)	(Torr)	(Watt)	4MS	NH3	He	N2
5.2%	350	4.2	650	200	1860	1550	250
6.4%	350	4.2	650	250	1860	1550	250
6.9%	350	4.2	650	270	1860	1550	250
7.6%	350	4.2	650	300	1860	1550	250
8.0%	350	4.2	650	320	1860	1550	250
8.5%	350	4.2	650	340	1860	1550	250

**Table 2:** ZnO RRAM characteristics with and without the SiCN film as an oxygen reservoir.

RRAM with	Set Voltage [V]	Reset Voltage [V]	On/Off Ratio	Endurance [Cycles]
ZnO Only	−5.26	3.37	127	<10
ZnO with SiCN	−1.3	1.125	69	53

## Data Availability

The data presented in this study are available on request from the corresponding author. The data are not publicly available due to related patent.
